# 5G/B5G mmWave Cellular Networks with MEC Prefetching Based on User Context Information †

**DOI:** 10.3390/s22186983

**Published:** 2022-09-15

**Authors:** Kazuki Maruta, Hiroaki Nishiuchi, Jin Nakazato, Gia Khanh Tran, Kei Sakaguchi

**Affiliations:** Tokyo Institute of Technology, Tokyo 152-8552, Japan

**Keywords:** 5G, beyond 5G, millimeter-wave, heterogeneous network, multi-access edge computing, prefetch, user context, backhaul limitation

## Abstract

To deal with recent increasing mobile traffic, ultra-broadband communication with millimeter-wave (mmWave) has been regarded as a key technology for 5G cellular networks. In a previous study, a mmWave heterogeneous network was composed of several mmWave small cells overlaid on the coverage of a macro cell. However, as seen from the optical fiber penetration rate worldwide, it is difficult to say that backhaul with Gbps order is available everywhere. In the case of using mmWave access under a limited backhaul capacity, it becomes a bottleneck at the backhaul; thus, mmWave access cannot fully demonstrate its potential. On the other hand, the concept of multi-access edge computing (MEC) has been proposed to decrease the response latency compared to cloud computing by deploying storage and computation resources to the user side of mobile networks. This paper introduces MEC into mmWave heterogeneous networks and proposes a content prefetching algorithm to resolve such backhaul issues. Context information, such as the destination, mobility, and traffic tendency, is shared through the macro cell to the prefetch application and data that the users request. Prefetched data is stored in the MEC and then transmitted via mmWave without a backhaul bottleneck. The effectiveness is verified through computer simulations where we implement realistic user mobility as well as traffic and backhauling models. The results show that the proposed framework achieved 95% system capacity even under the constraint of a 1 Gbps backhaul link.

## 1. Introduction

Since the emergence of new applications, mobile networks have shifted from being centric on the mobile phone to many types of devices, such as wearable and machine-type devices. The global mobile data traffic is expected to be increased at an annual average growth rate of 46%, and, in 2022, was seven times as much as in 2017 [1]. To address this challenge, the millimeter-wave (mmWave) frequencies have been allocated in the fifth generation (5G) and beyond 5G (B5G) [2]. Currently, mmWave bands from 24 to 40 GHz are available as FR2. Use of the 60 GHz unlicensed band, i.e., new radio-based access to the unlicensed spectrum (NR-U), is also under consideration in release 17 as further extension towards B5G [3,4].

Here, the mmWave has two key characteristics; high-throughput performance due to the spectral resources available and short coverage due to large path attenuation. To obtain the mmWave coverage everywhere, a heterogeneous network using both mmWave and Sub-6 GHz (mmWave HetNet) has been proposed [5,6]. The mmWave HetNet adopts a network architecture where many mmWave small-cell base stations (BS) are overlaid on the conventional macro cell operated at Sub-6 GHz.

In addition, C/U splitting, in which the macro cell BS is always in charge of the control plane (C-plane), has been proposed to prevent instantaneous disconnections due to frequent handovers. This network architecture enhances the system capacity 1000 times [5]. One of the critical challenges for realizing mmWave HetNet is a limitation of the backhaul capacity. Although the mmWave access can provide excellent throughput of several Gbps, a large capacity and expensive backhaul link, such as 10 Gbps, is required to demonstrate its capability in all small-cell sites.

According to the worldwide optical fiber line penetration rate [7], there are few countries where sufficient backhaul lines are established other than Japan and South Korea. In the case of a mmWave system being operated under such a low capacity backhaul line, it becomes a bottleneck, and it is impossible to expect a high system rate as an end-to-end system. From the above background, this paper proposes multi-access edge computing (MEC)-enabled content prefetching based on the user context information to fully leverage the mmWave capability.

MEC technologies [8,9,10] have been investigated in Beyond 5G (B5G) networks that distribute computation and storage capability to the edges of network. This enables offloading computational tasks produced on UE to the MEC, executing heavy processes (e.g., machine learning and games), and saving power consumption on user equipment (UE). Additionally, by installing a virtual machine/container instance of a server in the MEC in advance, users can access the application and obtain data with low latency compared to the cloud when requesting it.

Prefetching has also long been studied for the purpose of speeding up web access. Its major challenge is prediction. It has been studied from a variety of approaches, e.g., content-based [11,12], spatio-temporal locality [13], user behavior [14], wireless perspective [15], and so on. Prefetching is especially effective in extreme networks, such as satellite communication where the transmission latency is significant [16].

As mobile data communications have become more mainstream, such as 5G, their target has shifted to real-time content, such as streaming video [17]. In addition, the emergence of MEC with the 5G mobile network is the best match for those prefetching [18], which is why this research area is still growing. In recent years, machine-learning based prefetching has been applied on 5G networks, to predict segment requests, bitrate and user-BS association [19]. The literature [20] investigated the caching performance of MEC-enabled small-cell networks considering the backhaul capability. It should be noted that caching is a technique to reuse already visited data. The technical meaning differs from prefetching, which attempts to cache un-visited data before use.

Cellular networks have made remarkable progresses in wireless access rates, and their true performance must now be considered in conjunction with the capacity of the backhaul network. The literature [21] analyzed the throughput performance of a capacity-constrained heterogeneous network where macro and small cells are intensively densely deployed with sharing microwave-band frequency resources.

The work in [22] optimized macro/small-cell associations to minimize the packet delay. Limitations in backhaul are considered to be only the transmission latency. The impact on limited backhaul in machine-type communication scenarios was investigated in [23], where only 4G-based macro cells were supported. The authors in [24] tackled progressive resource allocation optimization on mmWave-integrated access and backhaul (IAB) scenario where wired/wireless systems co-exist. The performance of backhaul constrained cellular networks has also been investigated with the content prefetching approach.

Content caching and content delivery were jointly optimized in ultra-dense small-cell networks with constrained-backhaul links [25]. This study assumed microwave-band small cells, and thus the impact of the backhaul constraint was relatively relaxed compared to high-capacity mmWave access. The work in [26] proposed a mobility-aware distributed pre-caching strategy for a content-centric vehicular network. Some chunks of the content were distributed to road-side units according to the vehicles’ transition probability. The effectiveness was evaluated in terms of the delay time and required backhaul traffic.

To realize dynamic content prefetching in wide cellular coverage with mobility prediction, multiple edge coordination is inevitable. Although the recent work in [27] proposed 5G network structure and sequences to support MEC application mobility, it only presented the methodology and did not reveal detailed performance analysis results. The above key contributions related to the content prefetching and cellular network under constrained backhaul are summarized in Table 1.

This work is motivated by the above background; incorporating mmWave into MEC is expected to enhance both the throughput and latency capabilities, even with limited backhaul capacity. However, its characterization has not been fully disclosed thus far. Our conference paper [28] investigated the fundamental effectiveness of the prefetching and mmWave HetNet where 60 GHz small cells were overlaid on macro cellular systems.

This paper is an extension of that study to analyze the impact of forthcoming 5G NR based MEC prefetching that uses a 28 GHz band. The 60 GHz band is expected to be additionally assigned as the B5G spectrum. This paper also discloses that the 60 GHz band is more effective in MEC prefetching in terms of the system capacity, access delay, and energy efficiency compared to the 28 GHz band. Our previous work [29,30] experimentally demonstrated the effectiveness of MEC prefetching in a simple environment. Other works [31,32] discussed the effectiveness of MEC from the market perspective involved with private 5G operators, under the requirement of end-to-end latency and backhaul capacity constraints. The key contributions and novelty of this work are summarized as follows:

- MEC-assisted demand traffic prefetching is introduced to fully utilize the potential of mmWave communication under the constraint of limited backhaul.
- The proposed prefetching algorithm is based on the user context information, such as the users’ position information, preferences, and so on. Our algorithm assumes that it can predict their destination and demand traffic amount to be delivered to the nearest edge server in advance of their arrival.
- The validity of the proposed scheme is elucidated through a sophisticated computer simulation, which accurately modeled the user mobility and association/handover to macro/small cells.

The rest of this paper is organized as follows. Section 2 describes the system architecture of mmWave Edge Cloud with its network topology and newly developed user mobility and traffic models to describe realistic scenarios. Section 3 proposes a prefetching algorithm to enhance both the user-experienced data rate and latency. Numerical evaluation results are presented in Section 4 to confirm effectiveness of the proposed mmWave Edge Cloud with prefetching algorithm. Finally, Section 5 concludes this paper.

## 2. System Model

**Figure 1:**
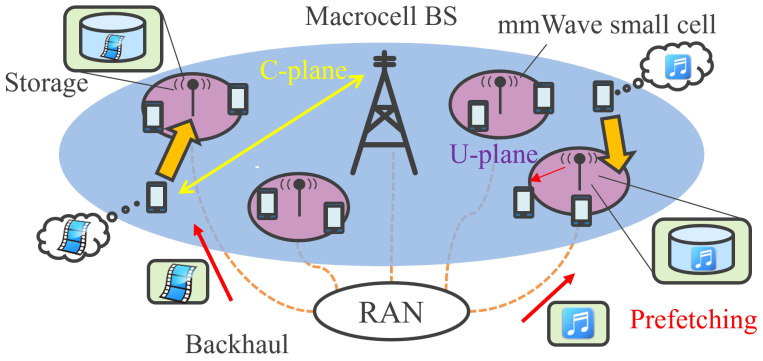
mmWave Edge Cloud system architecture.

We extended the system level simulator of mmWave HetNet developed in [5] to assess the effectiveness of combining mmWave access and MEC in the case of limited backhaul capacity. For that purpose, we developed a new mobility model, traffic model, and prefetching algorithm. Figure 1 shows the schematic overview of our proposed mmWave Edge Cloud 5G cellular system.

### 2.1. mmWave Heterogeneous Network

In this research, we consider HetNet based on the centralized radio access network (C-RAN) architecture [33] where mmWave small cells are overlaid on conventional LTE macro cells and they can tightly interwork. mmWave communication is assumed to be 5G NR in the 28 GHz band and NR-U [4] in the 60 GHz band, which includes coordination with IEEE802.11ad/ay standards [34], etc. The system performance is evaluated at the center macro cell surrounded by six interfering macro cells. Each small-cell BS is installed at the center of hotspot area and has three-sector mmWave access, a wired backhaul line, and Edge Cloud storage. This paper assumes only one sub-channel among the four sub-channels of 802.11ad to be used commonly at all the three sectors of each small-cell BS. In each sector, transmit signal is steered to the desired user by beam forming protocol using massive antennas equipped in mmWave small-cell BSs.

In addition, the mmWave Edge Cloud system has a novel C-plane, which can gather abundant user context information, such as mobility, traffic generation time, traffic quantity, and traffic type. This paper assumes that the network (macro/small-cell BSs) can acquire the context information of UE perfectly and that the UE handsets have dual connectivity to both small and macro cells. Based on the context information, if a UE’s demand traffic is small or of a type that cannot be stored in the edge storage, a macro cell BS will take charge of the UE’s U-plane.

Otherwise, if demand traffic is large and of a type that can be stored at the edge, the data can be gradually transmitted and stored in advance at the Edge Cloud storage, which is installed at the site of mmWave small-cell BS. This procedure is defined as ‘prefetching’. Prefetching can reduce or zero out the user-experienced backhaul latency and enables communication through high-speed mmWave access even under the constraint of low-capacity backhaul.

### 2.2. User Distribution and Mobility

A new user mobility model is developed for facilitating evaluation of our proposed network with MEC. The conventional hotspot model defined by 3GPP [35] merely specifies a ratio of the number of hotspot and non-hotspot users within the macro cell and, thus, does not take user mobility into account. To circumvent that issue, this paper extends the 3GPP hotspot model via introducing a proper mobility model. More specifically, there are several hotspots in the target macro cell, and users move at a constant speed within the macro cell partitioned into grids of regular distance. The user movement algorithm is as follows:

i. The user selects one hotspot area randomly as a destination when entering the target macro cell.
ii. The user moves to the destination and stays there for a certain time.
iii. After staying, the user goes outside of the macro cell.
iv. The user enters the macro cell as a new user, and then leaves the macro cell repeatedly until the end of the evaluation time.

**Figure 2:**
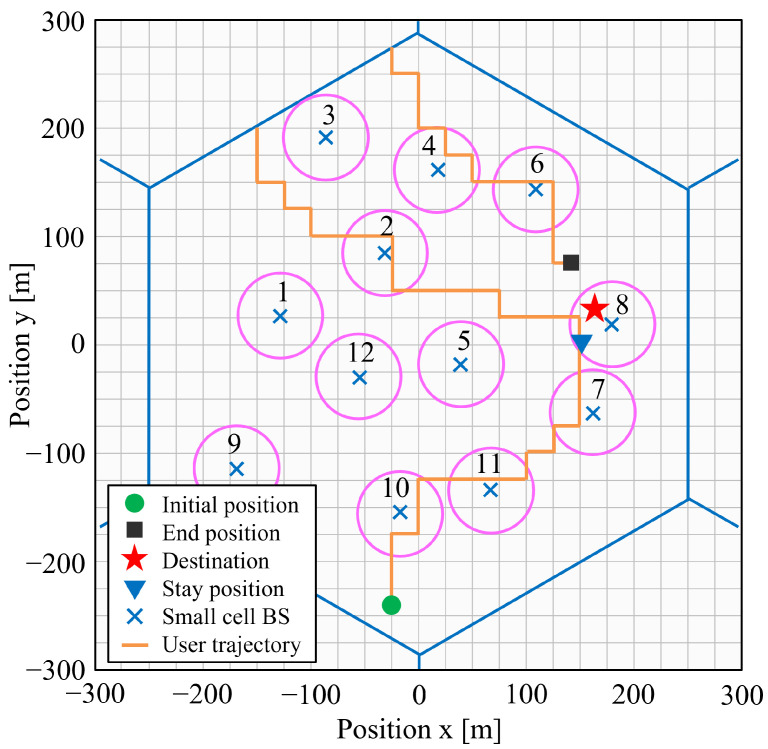
The user mobility model.

**Figure 3:**
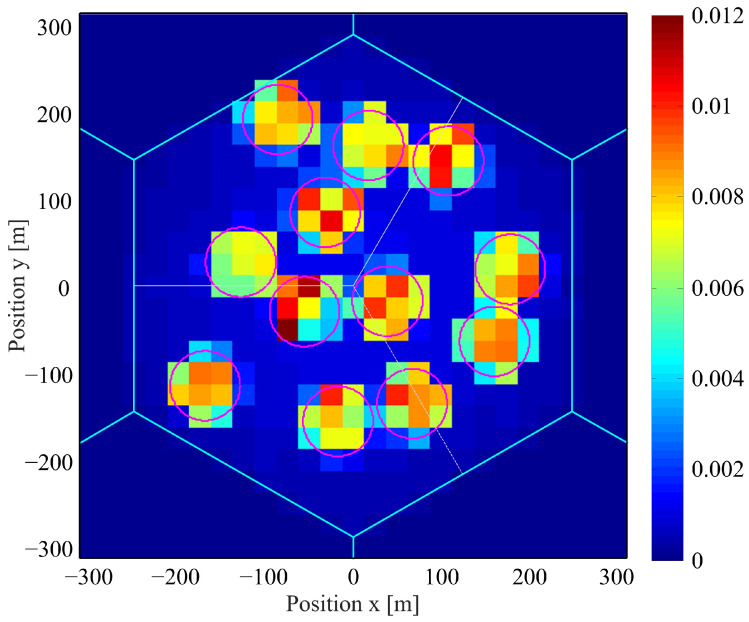
The spatial distribution of users.

As explained in the fourth step, this algorithm is repeated. Figure 2 shows the actual movement of one user in this simulation. The user moves from the initial position (circle) to the end position (square). The destination (star) in this figure is the final destination, which changes depending on the state of whether the user goes to the hotspot area or outside of the macro cell. The user stays at a nomadic position (triangle). Table 2 shows the parameters of our mobility model. Figure 3 shows the spatial distribution of users. The color bar in the figure represents the probabilistic density, and the totalization of these values within and out of hotspot areas will provide exactly the preferred hotspot ratio as defined by 3GPP.

### 2.3. Traffic Model

The traffic model should be associated with the mobility model. That is, users might require a large amount of traffic in the hotspot area defined in the mobility model. This assumption is reasonable since nomadic (or static) users usually demand large traffic (e.g., video data and backup), contrarily to mobility users who request small traffic (e.g., mail, voice call). Regarding our considered traffic distribution, the traffic quantity is based on measurement data [36] fitted with a gamma distribution, and the average parameters are multiplied by a factor of 1000 in anticipation of the traffic increases in the next 10 years.

**Figure 4:**
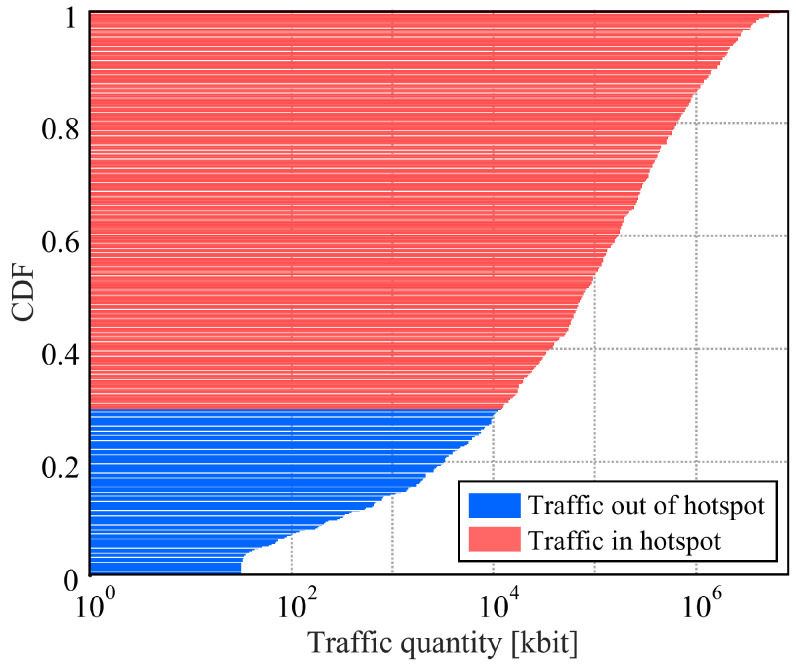
The CDF of the traffic amount to occur.

Figure 4 shows the traffic distribution of one user where the traffic colored red is generated inside hotspots, while blue traffic is generated outside of hotspots. In this paper, we assume that all types of generated traffic can be held in storage. The traffic generation interval follows an exponential distribution. The requested traffic is kept until it is sent or reaches the timeout. Table 3 shows the parameters with respect to the traffic model.

## 3. Prefetching Algorithm

Prefetching is to store the data, which is predicted for the UE demand, at the small-cell BS prior to their request. Small-cell BSs serve as the edge cloud. In order to perform prefetching, it is necessary to consider which small-cell site should be selected for storing the users’ demand and when to prefetch. In this paper, only the heavy traffic marked as red in Figure 4 will be prefetched. For this purpose, we developed the following algorithm with two steps.

### 3.1. Process for Data Prefetching

**Figure 5:**
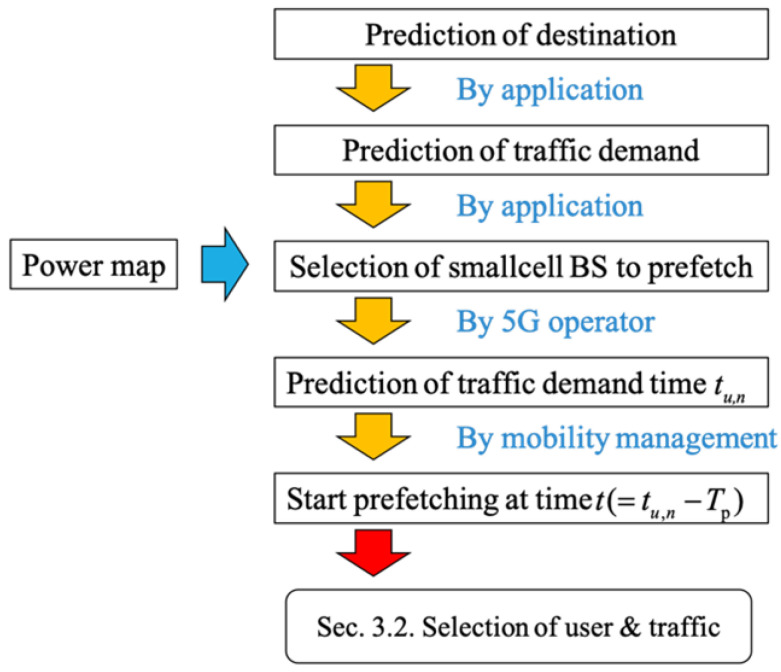
The pre-association process.

**Figure 6:**
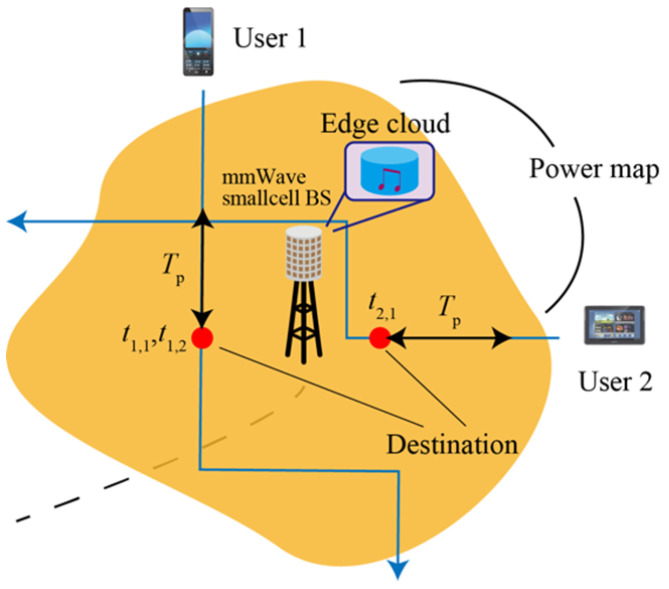
Illustration of pre-association.

First, we consider which small-cell BSs should be selected to prefetch the UE data. It needs to know the UE context information, such as the destination, traffic demand, and mobility. Figure 5 presents the flowchart of how to collect and use such context information in the pre-association process, while Figure 6 visualizes this process. First, the UE destination is assumed to be predicted by specific applications, e.g., the UE’s private calendar/agenda. This application will also predict the type and amount of traffic that the UE will demand at the predicted destination.

In the next step, based on the information about the UE destination, the 5G operator will predict its received Signal-to-Interference-plus-Noise Ratio (SINR) using a power map measured in advance (e.g., at the time of deployment of small-cell BSs) and pre-associate the UE to the small-cell BS, which yields the highest SINR to the UE for facilitating the prefetching process as explained in the next step. When UE is close to the destination, the 5G operator’s mobility management function will track the UE movement and predict the time $t_{u,n}$ that the UE will arrive at the destination and request the *n*-th traffic. If $t_{u,n}$ is smaller than $T_{p}+t$, which is a design parameter called the prefetching window in this paper, the future demand traffic will be prefetched. Since there will be different traffic requests from many UE within this prefetching window, a prefetching algorithm for proper scheduling is required, which will be explained in the following section. This paper assumes that context information can be predicted perfectly.

### 3.2. Objective Function Including User Context Parameters

**Figure 7:**
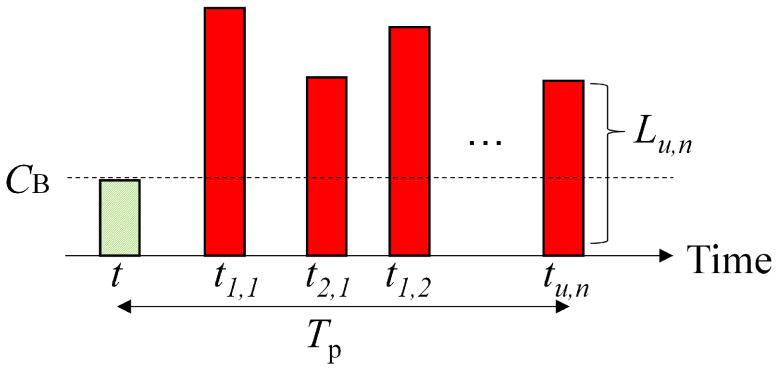
Illustration of traffic generation.

Since a common backhaul line is used for prefetching all UE traffic associated with a specific small-cell BS, we need an algorithm to decide the scheduling order on the backhaul line. In other words, we consider which *n*-th traffic of the *u*-th user should be selected to allocate backhaul resource $C_{B}$ at time instant *t*. Figure 7 illustrates the procedure of traffic generation when focusing on a small-cell BS. We utilize the weighted proportional fairness (WPF) scheduling method [37]. The objective function $O_{u,n}\left(t\right)$ for the *n*-th traffic of the *u*-th user at time instant *t* can be defined as:

(1) $$\begin{array}{c} O_{u,n}\left(t\right)=\frac{L_{u,n}}{B_{u}\left(t\right)}w_{u,n}^{\alpha }\left(t\right), \end{array}$$

where $L_{u,n}$ is the total traffic generated (in the future) for the *u*-th user with *n*-th traffic. The larger $L_{u,n}$ enlarges the value of the objective function $O_{u,n}\left(t\right)$. This indicates that large traffic needs to be prefetched at a higher priority to take full advantage of high-speed mmWave access. $B_{u}\left(t\right)$ is the accumulated backhaul resource allocated to the *u*-th user up to time *t*. α is defined as the PF coefficient. $w_{u,n}\left(t\right)$ is the timing weight taking into account the traffic generation time for the *n*-th traffic of the *u*-th user at time instant *t*, which can be expressed as:

(2) $$\begin{array}{c} W_{u,n}\left(t\right)=\frac{T_{p}}{t_{u,n}-t}, \end{array}$$

where $t_{u,n}$ is the time instant when the *u*-th user will demand the *n*-th traffic in future. In short, at the time *t*, values of the objective function, $O_{u,n}\left(t\right)$, and the timing weight, $W_{u,n}\left(t\right)$, are enlarged as the traffic generation time becomes closer. Finally, one *u*-th user and the corresponding *n*-th traffic is selected at time *t*, maximizing $O_{u,n}\left(t\right)$ among all users and the total traffic within the target prefetching duration $T_{p}$.

(3) $$\begin{array}{c} \left\{u(t),n(t)\right\}=\underset{u,n}{\arg\;\max}\;O_{u,n}\left(t\right). \end{array}$$

Backhaul resource $C_{B}$ is allocated to the n(t)-th traffic of the u(t)-th user and the corresponding data is stored in the Edge Cloud storage.

## 4. Computer Simulation

### 4.1. Simulation Condition and Performance Metric

We evaluated the performance of the mmWave Edge Cloud system through numerical simulation. Table 4 lists the simulation parameters. The macro-cell BS and the small-cell BS conform to the standard specifications of 3GPP (2.1 and 28 GHz) and IEEE802.11ad (60 GHz). The installation of the 60 GHz band is positioned as a performance evaluation for future B5G cellular networks, such as NR-U. The BS antenna beam pattern follows the 3GPP and IEEE802.11 specifications [34,38]. Channel model is based on the quasi deterministic radio channel generator (QuaDRiGa) [39], which provides detailed representation of spatial correlation characteristics in a MIMO channel. Line-of-sight (LOS) component varies with the distance between the BS and the UE. It is modeled as a stochastic component in macro [38] and small cells [40], respectively. For user association, we apply the multi-band cell association method presented in [41]. The system rate, *R*, combining the transmission rates of macro and small cells, is defined as follows.

(4) $$\begin{array}{cc} R & =E\left[\sum_{j_{M}=1}^{J_{M}}\sum_{u\in M_{j_{M}}}\min\left(\frac{W_{M}C_{u,j_{M}}}{|M_{j_{M}}|},\frac{L_{u}^{\mathrm{rem}}}{T_{s}}\right)+\sum_{s=1}^{N_{S}}\sum_{j_{S}=1}^{J_{S}}\sum_{u\in S_{s,j_{S}}}\min\left(\frac{W_{S}C_{u,s,j_{S}}}{|S_{s,j_{S}}|},\frac{D_{u,s}^{\mathrm{rem}}}{T_{s}}\right)\right], \end{array}$$

where $W_{M}$ and $W_{S}$ denote the available bandwidth at macro cells and small cells, respectively. $C_{u,j_{M}}$ and $C_{u,s,j_{S}}$ are the link capacity for the *u*-th UE and *s*-th small cell. $T_{s}$ is the time slot. $L_{u}^{\mathrm{rem}}$ is the instantaneous remaining traffic demand of the *u*-th UE. $N_{s}$ is the total number of small-cell BSs. $J_{M}$ and $J_{S}$ are the number of sectors at macro cell BS and small-cell BS. $M_{j_{M}}$ is the set of users belonging to a sector $j_{M}$ of macro cell BS, and $S_{s,j_{S}}$ is the set of UE belonging to a sector $j_{S}$ of small-cell BS *s*. $D_{u,s}^{\mathrm{rem}}$ is the data stored in storage for the *u*-th UE and the *s*-th small cell, which is expressed in detail as:

(5) $$\begin{array}{c} D_{u,s}^{\mathrm{rem}}=\min\left(C_{B}T_{u,s},L_{u}^{\mathrm{rem}}\right), \end{array}$$

where $T_{u,s}$ is the total time slot for the *u*-th UE at the *s*-th small cell determined by the proposed prefetching algorithm—namely, mmWave high-speed access is released from low backhaul limitation $C_{B}$ and accordingly the system rate, *R*, can be improved due to the prefetching. On the contrary, if the total time slot $T_{u,s}$ is small, mmWave access cannot demonstrate its capability due to the bottleneck of low backhaul capacity.

### 4.2. Numerical Analysis

**Figure 8:**
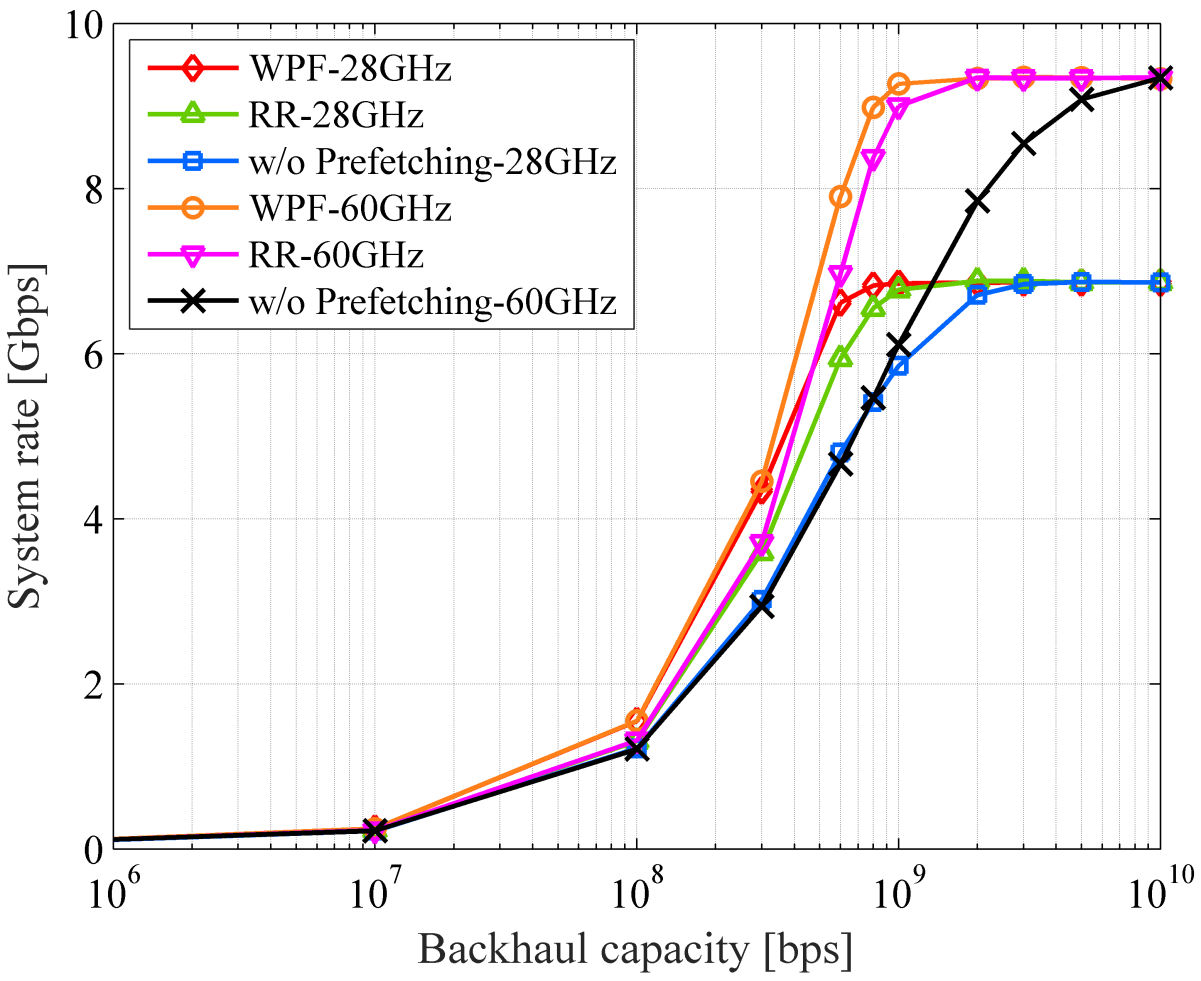
The system rate vs. the backhaul capacity.

Figure 8 plots the system rates for the 28 and 60 GHz mmWave bands against the backhaul capacity with several prefetching algorithms: WPF, Round-Robin (RR), and without prefetching. In the case of backhaul capacity with 0 bps, the system rate approaches that of the macro cell—it is about 100 Mbps. On the other hand, unlimited backhaul capacity, such as 10 Gbps, diminishes the effectiveness of the prefetching. The prefetching can enhance the system rate under the constraint of the backhaul capacity. Several existing works (e.g., [42,43]) have shown the high capacity provided by mmWave. In this study, we impose a backhaul constraint on it, resulting in the “w/o prefetching” characteristics shown in the figure.

By applying prefetching, the demand traffic can be appropriately allocated to the limited backhaul resources and immediately delivered to the end user; it can fully leverage the capability of the millimeter wave. The prefetching is effective up to a 3 Gbps backhaul capacity for the 28 GHz band and 10 Gbps capacity for the 60 GHz band. The 60 GHz band requires larger backhaul links due to its higher access rate, which allows more benefit from prefetching. The proposed prefetching algorithm can further improve system rate compared to an RR-based one.

With the 60 GHz band, the proposed WPF with 1 Gbps-backhaul can achieve about 95% of the maximal system rate, which is conventionally realized by 10 Gbps-backhaul. Although there are various algorithms and stochastic constraints for prefetching and mobility prediction as introduced in [11,12,26], they are treated as ideal in this study. This point needs to be evaluated in a more detailed manner, and we would like to discuss it in our future work.

**Figure 9:**
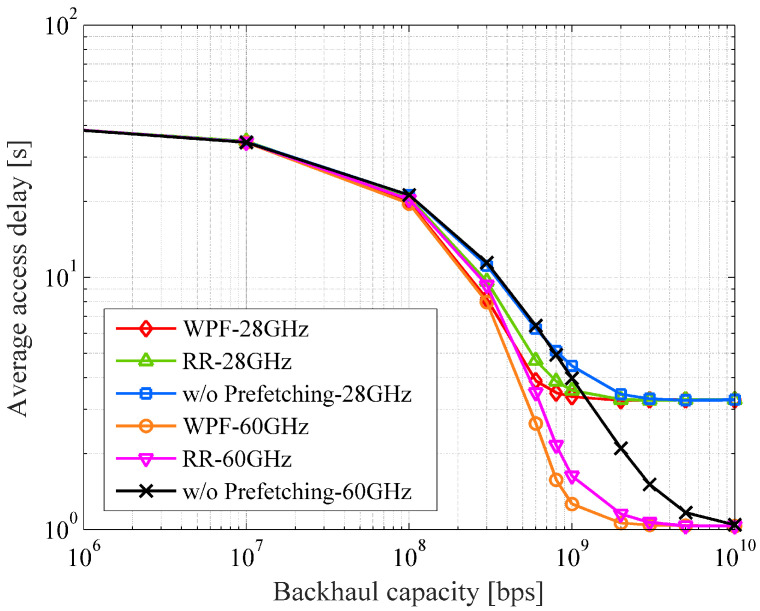
The average download time vs. the backhaul capacity.

Figure 9 shows the average download time with the backhaul capacity. This is measured by the total time required to completely transfer a UE’s demanded traffic. The maximum download time in the case of only macro cell is about 40 s. For the backhaul capacity of 1 Gbps, the proposed prefetching algorithm can reduce the delay by factors of approximately 67% and 26% in the 28 and 60 GHz bands, respectively, as compared to the conventional case without MEC and prefetching.

**Figure 10:**
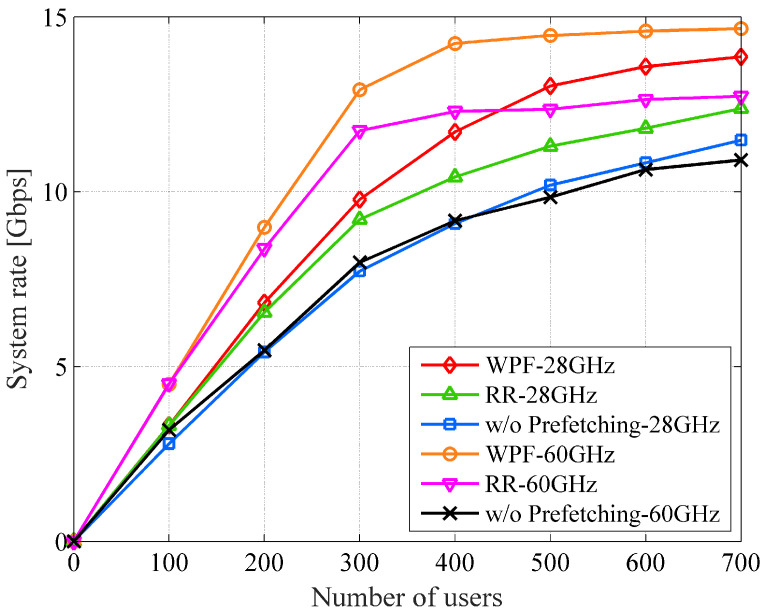
The system rate vs. the number of users (1 Gbps backhaul capacity and 500 s time window).

Figure 10 shows the system rate against the number of users with backhaul capacity of 1 Gbps and a time window of 500 s. The system rate increases linearly and drastically from 0 to 300 users because this network system has surplus communication resources, and the congestion of demand traffic rarely happens in the region. From 300 to 500 users, congestion of the traffic demand starts due to the shortage of resources, and the system rate increases slowly and tends to saturate. From 500 or more UE, the system reaches the upper limits. Comparing two prefetching algorithms and conventional one, the gap of system rate among them tends to be enlarged as the number of users increases; the proposed approach becomes more advantageous. In short, in an environment where many users exist, the usefulness of the prefetching including our proposed algorithm could be more important.

**Figure 11:**
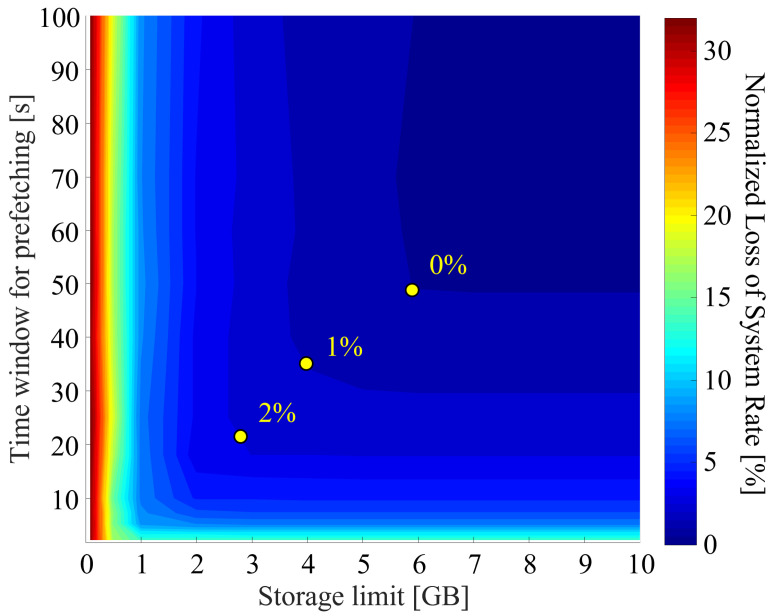
Normalized loss of the system rate (1 Gbps backhaul capacity, 60 GHz access, and WPF with 200 UE).

Figure 11 shows the normalized loss of the system rate against different storage limits and time windows on the conditions of 1 Gbps backhaul capacity with 60 GHz band, WPF algorithm, and 200 UE. The loss of system rate is defined as a ratio against the maximum system rate. In the figure, the colors show the loss level, and the boundaries are drawn at every 1% of loss. Essentially, the storage limit and the time window should be smaller in terms of the installation cost and prefetching performance.

Particularly concerning the latter, a small time window can prevent a communication performance deterioration from mis-prefetching if the context information contains an error. However, as shown in the figure, setting both values too small may result in non-negligible degradation of the system rate. A large amount of data cannot be stored in an overly limited storage capacity, and prefetching cannot be conducted in time for demand traffic in too short a time window. Therefore, appropriate values should be chosen to fully obtain the benefits of prefetching. For example, we recommend selecting 4 GB and 35 s as the storage and time window, which provides only 1% loss from the ideal case.

**Figure 12:**
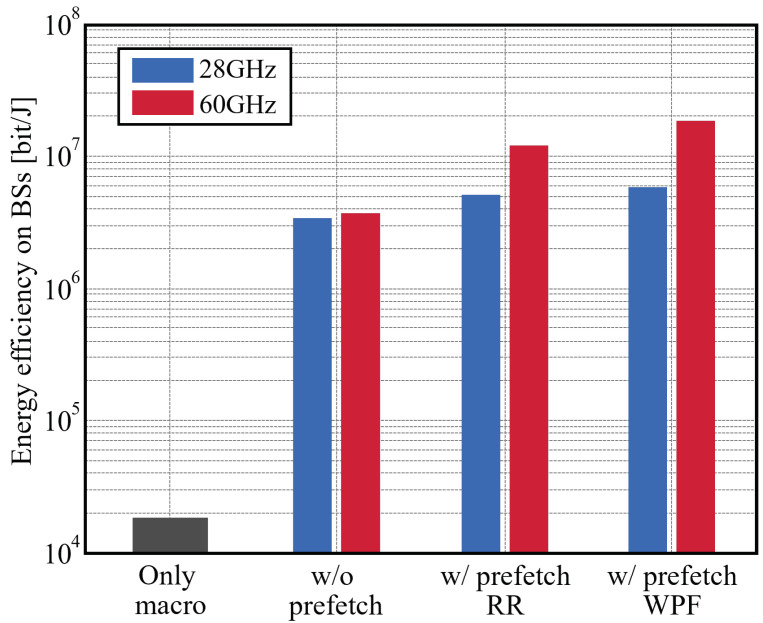
The energy efficiency (1 Gbps backhaul capacity, 200 UE).

In addition, mmWave edge cloud is beneficial from the viewpoint of power consumption. The energy efficiency of the network $\eta_{P}$ (bit/J) is defined as:

(6) $$\begin{array}{c} \eta_{P}=\frac{L}{T_{M}P_{M}+T_{S}P_{S}}, \end{array}$$

where *L* [bit] is the total traffic size occurring in the network, $T_{M}$ and $T_{S}$ [s] are the total time duration that the macro cell and small-cell BSs consume to transfer data. $P_{M}$ and $P_{S}$ [W] denote the power consumption, i.e., the transmit power in macro/small-cell BSs as shown in Table 4. Figure 12 shows the comparison of the energy efficiency $\eta_{P}$ in four cases. Employing mmWave small cells can significantly improve the energy efficiency compared to the case with only macro cells. This is because the mmWave can deliver more traffic data in a shorter communication duration. Furthermore, introducing MEC assisted by prefetching is more effective in saving power consumption.

In the 60 GHz band, energy efficiency of the RR-based prefetching is 3.2-times higher than the case without prefetching, and improvement of the proposed WPF is enhanced 5.0 times. In 28 GHz band, the improvement is small but still effective; the RR-based and proposed prefetching schemes attain 1.5- and 1.7-times higher energy efficiency compared with the conventional one.

### 4.3. Discussion and Future Work

In our future works, the two following issues should be considered. First, to investigate the performance bound of the proposed algorithm, this paper assumed that context information can be given ideally by the network. However, this assumption is not so practical since, in many circumstances, context information cannot be extracted accurately due to many reasons, e.g., privacy issues and/or prediction errors. Performance evaluation taking prediction errors into account is one of our future works. Second, since this paper dealt with traffic scheduling at the backhaul separately from that at the access links, joint optimization at both links simultaneously might promise further system performance improvement, which will be investigated as an advanced issue.

TokyoTech is now constructing an outdoor private B5G and MEC testbed in Ookayama campus [44,45]. The feasibility of our proposed system will be validated through a full-scale proof-of-concept.

## 5. Conclusions

In this paper, we proposed a novel 5G/B5G cellular network architecture enabled by mmWave Edge Cloud incorporated with the data prefetching based on users’ context information. In order to fully leverage the potential of mmWave broadband communication, prefetching is essential under the constraint of the backhaul capacity. In our proposal, user demand is predicted by utilizing context information, such as the destination, communication history, and mobility. Furthermore, the prefetching data amount is determined according to the weighted PF algorithm. Simulation evaluation confirmed the effectiveness supposing mmWave small cells operated at the 28 or 60 GHz bands.

Our results clarified that the proposed prefetching with WPF can maintain about 95% of the maximum system rate and can reduce about 67% of the average download time, even if the backhaul capacity is constrained to 1 Gbps. Therefore, the proposed scheme, including the overall system concept, could greatly contribute to the true realization of B5G. Our future work includes introducing a more practical model for mobility prediction with context information as well as joint optimization of access/backhaul resource assignment. Furthermore, a full-scale proof-of-concept will be conducted using an outdoor private 5G testbed.

## Figures and Tables

**Table 1:** Contributions to content prefetching and cellular network under constrained backhaul.

Year	Authors	Key Contribution
1998	Jiang et al. [11]	Web **content prefetching** based on access probability
2002	Shinkuma et al. [15]	Adaptive modulation and coding for access probability-based web **content prefetching**
2012	Nagase et al. [16]	Web **content prefetching** and acceleration for extremely large latency network
2016	Zhang et al. [22]	Delay-based macro/small-cell association under **limited backhaul**
2019	Jing et al. [25]	Joint optimization of **content prefetching** in microwave-band ultra-dense small-cell network with **limited backhaul**
2020	Esquivel-Mendiola et al. [23]	4G-based machine-type communication performance on **limited backhaul**
2020	Behravesh et al. [19]	DASH **content prefetching** by machine learning-aided prediction of user association and video segment
2021	Joo et al. [14]	Predictive web **content prefetching** utilizing user interaction
2022	Oh et al. [26]	Mobility-aware distributed proactive **content prefetching** strategy for vehicular network
2022	Maruta et al. (This Work)	MEC aided **content prefetching** to fully utilize **mmWave** Capacity with **limited backhaul** in macro/small-cell heterogeneous networks

**Table 2:** The mobility parameters.

Parameters	Values
Macro cell radius	250 m
Hotspot area radius	40 m
User speed	1 m/s
Road interval	25 m
Staying time	Exponential distribution of avg. 500 s

**Table 3:** The parameters for the traffic model.

Parameters	Values
Traffic quantity	Gamma distribution shape parameter k=0.2892 scale parameter $\theta =2.012\times 10^{5}$
Traffic bias	4 kbits
Occurrence interval	Exponential distribution of avg. 8 s
Timeout	60 s

**Table 4:** The simulation parameters.

Parameters	Values
Bandwidth (Macro/Small)	10 MHz/400 MHz/2.16 GHz
Carrier freq. (Macro/Small)	2.1 GHz/28 GHz/60 GHz
Number of BS (Macro/Small)	7 (1 for target)/84 (12 for target)
Number of BS sectors (Macro/Small)	3/3
BS antenna elements (Macro/Small)	4/128
US antenna elements	2
BS antenna height (Macro/Small)	25 m/10 m/4 m
UE antenna height	1.5 m
BS antenna beam pattern (Macro/Small)	3GPP-LTE [38]/5G-NR/IEEE802.11ad
UE antenna beam pattern	Half-wave dipole
Channel model	QuaDRiGa [39]
Tx power (Macro/Small)	46 dBm/10 dBm
LOS probability (Macro/Small)	[38]/[40]
Path loss model (Macro/Small)	[38]/[40]
Shadowing std. (Macro/Small)	[38]/[40]
Backhaul capacity	0 bps, 1 Mbps–30 Gbps
Prefetching window	0–100, 500 s
PF coefficient	3
Storage limitation	0–10 GB, Infinity

## Data Availability

Not applicable.

## Abbreviations

The following abbreviations are used in this manuscript:

mmWave	Millimeter-wave
5G	Fifth generation mobile communications system
B5G	Beyond 5G
NR-U	New radio-based access to unlicensed spectrum
HetNet	Heterogeneous network
BS	Base station
UE	User equipment
C-plane	Control plane
U-plane	User plane
MEC	multi-access edge computing
C-RAN	Centralized radio access network
3GPP	Third generation partnership project
SINR	Signal-to-interference-plus-noise power ratio
WPF	Weighted proportional fairness
RR	Round robin
